# Supplementary material for: Factors Affecting Attitudes towards COVID-19 Vaccination: An Online Survey in Slovenia
Source: Vaccines (Basel). 2021 Mar 12;9(3):247. doi: 10.3390/vaccines9030247 (PMC8002174; doi:10.3390/vaccines9030247)
Supplement: Supplementary file 1 [file vaccines-09-00247-s001.zip › Supplements corrected after review/Supplement 1 - Questionaire translation to English.docx]

**The attitude of Slovenes towards vaccinating against SARS-CoV-2 virus**

Dear Sir/Madam!

As students of the Faculty of Medicine, University of Maribor, we would like to request for your participation in an online survey on COVID-19 epidemic and on vaccination against
SARS-CoV-2 virus. The research is being carried out under the supervision of prof. Dr Matjaž Zwitter and the team of COVID-19 Sledilnik, which has helped us prepare the survey questionnaire. We kindly ask you to take 8 minutes of your time and fill in the questionnaire. The survey is intended to capture responses from anybody over the age of 15, participation is voluntary and there are no wrong answers - we are mainly interested in your personal view on the current situation. The answers are completely confidential and will be used for study purposes only. The results will be analysed and presented publicly in the media, as well as used for pedagogical and research purposes. When you complete the questionnaire, we kindly invite you to forward it to the people around you. You would especially be of great assistance if you help our study reach those who use internet less often. We appreciate your cooperation and great you warmly.

Students of the Faculty of Medicine, University of Maribor

**Q1 - With the following questions we would like to examine your experiences during the epidemic.**

_____________________________________________________________________

**Q2 - Have you ever been infected with the virus SARS-CoV-2?**
No.
Yes, the infection was affirmed.

**Q3 - Do you personally know anybody that has been infected with the virus SARS-CoV-2?**
No.
Yes.

**Q4 - Do you personally know anybody that has needed hospital treatment due to infection with the virus SARS-CoV-2?**
No.
Yes.

**Q5 - Do you personally know anybody that has died due to infection with the virus SARS-CoV-2?**
No.
Yes.

**Q6 - How much do you trust individual sources of information about SARS-CoV-2 vaccination?**

|  | do not trust at all | do not trust | neither do trust, neither do not trust | trust | completely trust |
| --- | --- | --- | --- | --- | --- |
| Reports on television and radio. |  |  |  |  |  |
| Daily newspaper. |  |  |  |  |  |
| National Institute of Public Health. |  |  |  |  |  |
| The ministry of health of the Republic of Slovenia. |  |  |  |  |  |
| World Health Organization (WHO). |  |  |  |  |  |
| The government of Republic of Slovenia. |  |  |  |  |  |
| Alternative explanations on social media. |  |  |  |  |  |
| Professional articles and research findings. |  |  |  |  |  |
| Expert opinion. |  |  |  |  |  |
| Information provided to me by acquaintances employed in the field of healthcare. |  |  |  |  |  |
| Information from friends and acquaintances that are not employed in the field of healthcare. |  |  |  |  |  |

**Q7 - Below you will find measures against the epidemic. Evaluate how irritating each of them individually was for you.**

|  | very irritating | slightly irritating | not irritating | measure does not apply to me |
| --- | --- | --- | --- | --- |
| Mandatory mask wearing. |  |  |  |  |
| Respecting the appropriate distance between people. |  |  |  |  |
| Restriction of movement to the municipality of residence. |  |  |  |  |
| Restriction of movement to country’s borders. |  |  |  |  |
| Restriction of movement in the night time. |  |  |  |  |
| Closure of stores (except supermarkets and pharmacies). |  |  |  |  |
| Closure of catering establishments. |  |  |  |  |
| Closure of cultural institutions. |  |  |  |  |
| Closure of kindergartens and schools. |  |  |  |  |
| Elimination of public transport. |  |  |  |  |
| Prohibition of private gatherings. |  |  |  |  |

**Q8 - Do you think that vaccination against SARS-CoV-2 virus should be mandatory for health workers and employees of nursing homes (with the exception of those who should not be vaccinated due to health reasons)?**

No.

Yes.

I do not know.

**Q9 - As the most important measure against SARS-CoV-2 virus epidemic vaccination is being planned all over the world – and is expected to be free of charge and voluntary. Are you planning to get vaccinated as soon as possible?**

I will definitely not get vaccinated.

I will probably not get vaccinated.

I will probably get vaccinated.

I will definitely get vaccinated.

**Q10 - Evaluate how much you agree with the statements below.**

|  | do not agree at all | do not agree | indecisive | agree | completely agree |
| --- | --- | --- | --- | --- | --- |
| I trust that the vaccine against SARS-CoV-2 virus is safe. |  |  |  |  |  |
| I believe that vaccination against SARS-CoV-2 virus is effective. |  |  |  |  |  |
| I would like to wait for more information on the safety of the vaccine against SARS-CoV-2 virus. |  |  |  |  |  |
| I am very scared of getting infected with SARS-Co-V2 virus. |  |  |  |  |  |
| I think that SARS-CoV-2 virus is equally dangerous as the influenza virus. |  |  |  |  |  |
| I have negative experiences with vaccinations – considering me or my loved ones. |  |  |  |  |  |
| Vaccination against SARS-CoV-2 virus is an attempt of controlling the population. |  |  |  |  |  |

**Q11 - What is your general opinion on vaccination against other infectious diseases?**

I am against all types of vaccines.

I only support vaccinations that are currently compulsory.

I support both compulsory and non-compulsory vaccinations (against HPV, tick-borne meningoencephalitis, influenza...).

**Q12 - Have you been previously vaccinated against the influenza virus?**

I have never been vaccinated.

I only get vaccinated occasionally.

Yes, I get vaccinated regularly (every year).

**Q13 - Would you like to add something more about SARS-CoV-2 vaccination that you haven’t been asked about?**

_______________________________

**Q15 - Gender:**

Woman.

Man.

**Q16 - Age:**

from 15 to including 24 years

from 25 to including 34 years

from 35 to including 44 years

from 45 to including 54 years

from 55 to including 64 years

from 65 to including 74 years

75 years or older

**Q17 - Level of completed education:**

I. or II. (incomplete or completed primary school)

III. or IV. (lower vocational or middle vocational education - 2/3 years)

V. (grammar school, secondary school)

VI. (high professional education)

VII. or VIII. (specialization in higher professional / university program, master's / doctorate)

**Q18 – Status:**
a student
self-employed or freelance
unemployed
retired
a farmer
a housekeeper
other:

**Q19 - Do you work in the healthcare field (nursing homes are also included)?**

No.

Yes, as a doctor.

Yes, as other personnel (nurse, healthcare technician, caregiver, physiotherapist, psychologist, social worker, occupational therapist).

Yes, as a medical student.

Yes, as a Nursing high school student or a faculty student

Yes, as a student of other fields.

**Q20 - Region you live in:**
Osrednjeslovenska.

Podravska.

Savinjska.

Dolenjska, Bela krajina in Kočevska.

Pomurska.

Gorenjska.

Posavska.

Koroška.

Obalno-kraška.

Goriška.

Primorsko-notranjska.

Zasavska.

**Q21 - Where do you live?**
In a city.
In the suburbs.
In a village.
