# Supplementary material for: Factors Affecting Attitudes towards COVID-19 Vaccination: An Online Survey in Slovenia
Source: Vaccines (Basel). 2021 Mar 12;9(3):247. doi: 10.3390/vaccines9030247 (PMC8002174; doi:10.3390/vaccines9030247)
Supplement: Supplementary file 1 [file vaccines-09-00247-s001.zip › Supplements corrected after review/Supplement 2 - letter to promote the survey.docx]

Dear Sir or Madam!
We are students of the Faculty of Medicine, University of Maribor. We have prepared this questionnaire titled “The attitude of Slovenes towards vaccinating against the SARS-CoV-2 virus” under the supervision of Dr Matjaž Zwitter and the team of COVID-19 Sledilnik.

In these dire times, we must stand together and help each other as best we can. Therefore, we kindly ask you to share this questionnaire by posting its link on your website or Facebook page. The link is: <https://1ka.arnes.si/cepivo>.

With your help we can reach a high number and a wide variety of people. We truly hope to achieve high-quality results which will be available to the public. Additionally, we would appreciate vast media coverage – and you can be part of this by spreading the word!

We thank you from the bottom of our hearts. Stay healthy and safe!

Rok Arh, Tina Gabrovec, Lucija Jazbec, Luka Petravić, Nika Rupčić, Nina Starešinič, Lea Zorman
