# Supplementary material for: Factors Affecting Attitudes towards COVID-19 Vaccination: An Online Survey in Slovenia
Source: Vaccines (Basel). 2021 Mar 12;9(3):247. doi: 10.3390/vaccines9030247 (PMC8002174; doi:10.3390/vaccines9030247)
Supplement: Supplementary file 1 [file vaccines-09-00247-s001.zip › Supplements corrected after review/Supplement 5 - additional tables.docx]

| Healthcare profession |  | Component 1: Trust in official sources | Component 2: Trust in alternative sources | Component 3: Distrust in government |
| --- | --- | --- | --- | --- |
| Non-HCP  (n = 9.972) | Mean | -0.04749 | 0.03499 | 0.00031 |
|  | Std. Deviation | 0.99892 | 1.00115 | 1.01779 |
| Physicians  (n = 770) | Mean | 0.63779 | -0.34752 | -0.12438 |
|  | Std. Deviation | 0.76918 | 0.88473 | 0.90951 |
| Other-HCP  (n = 674) | Mean | -0.23700 | 0.00688 | -0.05607 |
|  | Std. Deviation | 1.03637 | 1.06894 | 0.95015 |
| Medical students  (n = 311) | Mean | 0.57490 | -0.29792 | 0.25498 |
|  | Std. Deviation | 0.73001 | 0.82663 | 0.78850 |
| HC students  (n = 313) | Mean | -0.11705 | 0.02128 | 0.16345 |
|  | Std. Deviation | 0.92297 | 1.02747 | 0.86066 |
| Total  (n = 12.040) | Mean | 0 | 0 | 0 |
|  | Std. Deviation | 1 | 1 | 1 |

S5: Table 1

Computed mean component values for different HCP and non-HCP, calculations relating to figure 4.

S5: Table 2

Results of binary regression analysis for previous flu vaccinations

| PREVIOUS FLU VACCINATIONS | Coef. | Std. Err. | z | P>\|z\| | [95% Conf. Interval] | |
| --- | --- | --- | --- | --- | --- | --- |
| Gender = male | 0,151 | 0,026 | 5,830 | 0,000 | 0,101 | 0,202 |
| Age (1 = 15-24, 2 = 25 -34, ..., 6 = 65-74, 7 = 75+) | 0,207 | 0,018 | 11,490 | 0,000 | 0,172 | 0,242 |
| Education = high | -0,186 | 0,027 | -6,800 | 0,000 | -0,240 | -0,132 |
| Profession = physician | 1,366 | 0,069 | 19,850 | 0,000 | 1,231 | 1,500 |
| Profession = other HCP | 0,444 | 0,054 | 8,250 | 0,000 | 0,338 | 0,549 |
| Profession = medical student | 1,186 | 0,095 | 12,440 | 0,000 | 0,999 | 1,373 |
| Profession = HC student | 0,183 | 0,077 | 2,390 | 0,017 | 0,033 | 0,333 |
| Component 1: trust in official sources | 0,462 | 0,014 | 34,040 | 0,000 | 0,436 | 0,489 |
| Component 2: trust in alternative sources | -0,114 | 0,013 | -9,050 | 0,000 | -0,138 | -0,089 |
| Component 3: distrust in government | -0,046 | 0,013 | -3,660 | 0,000 | -0,071 | -0,022 |
| _cons | -0,551 | 0,037 | -14,750 | 0,000 | -0,624 | -0,478 |

S5: Table 3

Results of word enrichment for each cluster. Clusters were compared one against all. Only words with FDR less than 0.1 are reported or the top three words by p-value if there are many significant words.

| Cluster | Size | Significant words | p-value | FDR |
| --- | --- | --- | --- | --- |
| C1 | 14 (0.6 %) | obligatory | 1.1e-18 | 8.2e-15 |
| C2 | 16 (0.7 %) | thank | 2.0e-30 | 1.5e-26 |
|  |  | effort | 2.2e-05 | 0.08 |
| C3 | 16 (0.7 %) | scam | 2.1e-33 | 1.6e-29 |
|  |  | century | 3.1e-07 | 1.2e-03 |
|  |  | global | 5.3e-06 | 0.01 |
| C4 | 23 (1 %) | side | 1.0e-24 | 8.0e-21 |
|  |  | effect | 7.8e-21 | 3.0e-17 |
|  |  | long-term | 2.5e-07 | 6.4e-04 |
| C5 | 1019 (44 %) | vaccine | 1.8e-24 | 8.6e-21 |
|  |  | effect | 2.3e-24 | 8.6e-21 |
|  |  | vaccinate | 2.0e-18 | 5.2e-15 |
| C6 | 22 (0.9 %) | as soon as possible | 1.7e-31 | 1.3e-27 |
|  |  | sufficient | 3.6e-08 | 1.4e-04 |
|  |  | begin | 1.4e-06 | 3.6e-03 |
| C7 | 12 (0.5 %) | dangerous | 4.0e-20 | 3.0e-16 |
|  |  | flu | 1.1e-15 | 4.3e-12 |
|  |  | more | 6.2e-09 | 1.6e-05 |
| C8 | 13 (0.6 %) | isolate | 4.1e-22 | 3.1e-18 |
|  |  | succeed | 6.5e-18 | 2.5e-14 |
|  |  | virus | 5.2e-09 | 1.3e-05 |
| C9 | 9 (0.4 %) | voluntary | 4.1e-22 | 3.1e-18 |
| C10 | 1176 (50.6 %) | measure | 1.3e-10 | 9.5e-07 |
|  |  | trust | 6.4e-09 | 2.4e-05 |
|  |  | government | 1.6e-08 | 4.1e-05 |
