# Supplementary material for: Factors Affecting Attitudes towards COVID-19 Vaccination: An Online Survey in Slovenia
Source: Vaccines (Basel). 2021 Mar 12;9(3):247. doi: 10.3390/vaccines9030247 (PMC8002174; doi:10.3390/vaccines9030247)
Supplement: Supplementary file 1 [file vaccines-09-00247-s001.zip › Supplements corrected after review/Supplement 3 - CHERRIES compliant methods_LD.docx]

**Detailed methods description according to the CHERRIES checklist for reporting results of e-surveys**

This study is reported per the Checklist for Reporting Results of Internet E-Surveys (CHERRIES) [1].

A cross-sectional study was designed to study public opinion towards COVID-19 vaccination in Slovenia. The target population of the survey was Slovenian residents aged 15 or more. The survey language was Slovenian, and it was developed through a collaborative effort between the authors and the COVID-19 Tracker Slovenia community (https://covid-19.sledilnik.org/). We also consulted with the Governmental Work Group on COVID-19 to see which questions they deemed important. Literature research was conducted to enhance the possibility of comparison of our work with the works of others. The questionnaire was pretested on a small sample of people (n=17) before being fielded.

The survey had 11 closed-ended questions regarding vaccination opinion in general, attitudes towards different news sources, personal perception of the so-called lockdown orders and one open-ended question to entail everything a person might want to add (the part was not covered in the questions above). To better understand the results and identify beliefs of subgroups, we also included seven demographic questions. These questions entailed gender, age group, education level, work status, employment in the medical community, region and living surroundings (city, suburb, or village). There were five pages to our questionnaire. The number of questions per page never exceeded four questions, except for the demographic information that was all taken from one page. Adaptive questioning was not used and there was no randomisation nor alternation to the sequence of questions. The English version of the survey used is added as Appendix 2.

Two surveys were run in parallel, one on a nonprobability sample using social media to disseminate the survey link and one on a probability-based online survey panel “JazVem” owned by the marketing research company Valicon Ltd. The nonprobability survey was conducted with the One-Click Survey (v 20.12.03) tool, while the probability survey was done using the company’s survey software. In both cases, the questionnaire could be filled out in a browser and did not require any software installation. The survey participation was voluntary. There were no monetary incentives offered to the participants in the nonprobability sample, but members of the JazVem panel got cooperation points that can be transformed into rewards.

The first survey was a convenience sample and did not have a tangible sample frame. To address the potential preselection bias of the sample, we contacted a wide array of national and local media and other organisations (n=173) to disseminate the survey link on their social media pages and other communication channels. The survey was open to anyone who had the link. The invitation for it is added as Appendix 1. In the eleven days that the survey ran, from Thursday, December 17 to Sunday, December 27, 2020, there were 45.633 clicks on the survey link. In total, 18.277 started to respond, of which 1.982 completed it partially and 16.245 fully. The completion rate was 89%.

Authors promised to make results public through media and report on the findings to governmental bodies. The study was not brought to approval by Institutional Review Board as no personally identifiable information was required from people responding to the survey. Even though there was no personal information collected, rigorous access security to the data and administration of the questionnaire in the survey tool environment was in place, using personal credentials for access. A log of all actions is held for both data storage and the survey tool environment.

On the first page, the survey informed the participants about the estimated length (eight minutes), aim of the survey, data policy, investigator, and possibility to refuse participation and leave the survey with no repercussion. A participant could always leave the survey on any page, but the system still logged their answers. Respondents were able to change their answers up to the point of completing the last page. There was a back button on all pages. There was no summary on the end with all the responses. The survey tool used and the way of dissemination (convenience sampling) of our survey link only allows for a calculation of completion rate (number of users who finished the survey divided with the users who clicked on the first page of the survey).

In addition, Valicon Ltd. used our questionnaire on a probability sample of the members of their JazVem online survey panel, the largest in Slovenia. More than three-quarters of the JazVem members were included in the panel with probabilistic sampling from the population register, while the rest were self-selected. The participation rate in surveys done in the panel is about 60%. Its representativeness for the Slovenian population has been proven several times by comparing it to official statistics. The survey was run on Monday and Tuesday, 21^st^ and 22^nd^ of December, 2020, and in total, 516 panel members participated. Sampling, data collection and survey adjustments were provided to the researchers free of charge. In addition to the survey responses, the database also includes supporting information about the participating panellists (salary, lifestyle characteristics, etc.). As the probability sample did not cover a sufficient number of health care professionals, the manuscript is based on the results of the larger nonprobability sample and the probability sample is used only for validation.

Analysis of quantitative data was done with SPSS (release 27.0.0.0, RRID:SCR_019096), except for ordinal regression and mediation analysis that was done in STATA (release 15.0, RRID:SCR_012763). We analysed complete and incomplete questionnaires. Incomplete questionnaires were only used for frequencies analysis, whereas complete questionnaires were used for a more in-depth statistical study. The sample was described using frequency distributions and bivariate cross-tabulations, and correlations were computed for pairs of variables. To reduce the dimensionality of data for scale questions, we carried out factor analysis to compute components to be used in further analysis. Based on the literature review, we developed a theoretical regression model with the intention to vaccinate as the dependent variable, which we were able to manage with our survey data. We ran an ordinal regression model to estimate the effect of a set of independent variables on the intention to vaccinate; the model included gender, age, education, flu vaccinations, healthcare professions and PCA components. For significance, a value 0.01 was used, instead of 0.05, because of the large sample size to exclude possibility of trivial correlations [2]. We did not measure the time necessary for the completion of the questionnaire, nor did we discern inputs based on this data point. To achieve more representative results, data were weighted by gender, age, and region but the weight was not used in the analysis.

Analysis of textual data was performed using Orange Data Mining (release 3.27.1, RRID:SCR_019811) [3]. We analysed a subsample of the respondents who completed the free-form questionnaire, specifically 2320 answers (12 %). These also excluded error values (e.g., “Err 509) and answers shorter than five characters (most of these were a form of “no”).

The answers were preprocessed with lowercase transform, tokenization by words, lemmatization with UDPipe 2 lemmatizer [4], stopword removal, and removal of digits. TF-IDF transform was used for vectorization of documents. Clustering was performed with cosine distance and hierarchical clustering with Ward linkage. Large subgroups were further explored with another layer of clustering with the same parameters. We analysed the clusters with Chi2, Student’s t-test and ANOVA for determining the difference between groups (either a selected subgroup versus all of them or subgroups with each other). To determine the content of each group’s answers, we used a mixture of close and distant reading [5]. For distant reading, we used word enrichment to determine statistically significant words for each subgroup.

**Sources**

1. Eysenbach, G. Improving the Quality of Web Surveys: The Checklist for Reporting Results of Internet E-Surveys (CHERRIES). *J. Med. Internet Res.* **2004**, *6*, e34, doi:10.2196/jmir.6.3.e34.

2. Head, K.J.; Kasting, M.L.; Sturm, L.A.; Hartsock, J.A.; Zimet, G.D. A National Survey Assessing SARS-CoV-2 Vaccination Intentions: Implications for Future Public Health Communication Efforts. *Sci. Commun.* **2020**, *42*, 698–723, doi:10.1177/1075547020960463.

3. Demšar, J.; Curk, T.; Erjavec, A.; Gorup, Č.; Hočevar, T.; Milutinovič, M.; Možina, M.; Polajnar, M.; Toplak, M.; Starič, A.; et al. Orange: Data Mining Toolbox in Python. *J. Mach. Learn. Res.* **2013**, *14*, 2349–2353.

4. Straka, M. UDPipe 2.0 prototype at CoNLL 2018 UD shared task. In Proceedings of the Proceedings of the CoNLL 2018 Shared Task: Multilingual Parsing from Raw Text to Universal Dependencies; 2018; pp. 197–207.

5. Jänicke, S.; Franzini, G.; Cheema, M.F.; Scheuermann, G. On Close and Distant Reading in Digital Humanities: A Survey and Future Challenges. In Proceedings of the EuroVis (STARs); 2015; pp. 83–103.
